# Spectrum of appearances on CT and MRI of hepatic epithelioid hemangioendothelioma

**DOI:** 10.1186/s12876-015-0299-x

**Published:** 2015-06-19

**Authors:** Lisha Zhou, Min-Yi Cui, Juxin Xiong, Zhi Dong, Yanji Luo, Hui Xiao, Ling Xu, Kun Huang, Zi-Ping Li, Shi-Ting Feng

**Affiliations:** 1 Department of Radiology, The First Affiliated Hospital, Sun Yat-Sen University, Guangzhou, Guangdong China; 2 Department of Radiology, Hospital of Stomatology, Guanghua School of Stomatology, Sun Yat-Sen University, Guangdong, China; 3 Department of Radiology, The Third People’s Hospital of Dongguan City, Dongguan, China; 4 Department of Pathology, The First Affiliated Hospital, Sun Yat-Sen University, Guangzhou, China; 5 University of Western Australia, Perth, Australia

**Keywords:** Liver, Epithelioid hemangioendothelioma, Computed tomography, Magnetic resonance imaging

## Abstract

**Background:**

This study aims to analyze the computed tomography (CT) and magnetic resonance imaging(MRI) characteristics of hepatic epithelioid hemangioendothelioma (HEHE).

**Methods:**

Eleven patients with histopathologically confirmed HEHE via surgical excision or biopsy were included. Imaging findings of these 11 patients were retrospectively analyzed (CT images obtained from all patients and MR images from five patients). Patterns of growth, characteristics of distribution, density/signal features, patterns of contrast enhancement, and changes of adjacent tissues were evaluated.

**Results:**

HEHE is characterized by multiple lesions in the liver. HEHE could be further categorized as three types when considering patterns of growth: nodular type(5 cases), coalescent type(1 case) and mixed type(5 cases). In this study, a total of 312 lesions were detected, 214(74.3 %) of which were subcapsular. All lesions appeared as hypodense while round lower density were found within 10 lesions(<2 cm) on unenhanced CT images. On MRI, all lesions demonstrated low signal intensity on T1 weighted images and high heterogeneous signal intensity on T2 weighted images when compared to the normal liver parenchyma. Other imaging features included “lollipop sign”(6 cases) and capsular retraction(6 cases). On contrast-enhanced CT and MRI, lesions smaller than 2.0 cm mostly showed mild homogeneous enhancement (214/227, 94.3 %); lesions measuring 2.0–3.0 cm in diameter showed ring-like enhancement (16/53,30.2 %) and heterogeneous delayed enhancement (29/53,54.7 %); lesions larger than 3.0 cm demonstrated heterogeneous delayed enhancement (26/32, 81.3 %).

**Conclusion:**

The imaging findings of HEHE showed some typical imaging features and size-dependent patterns with contrast enhancement on both CT and MR images, these features can be used for accurate imaging diagnosis of HEHE.

## Background

Epithelioid hemangioendothelioma (EHE) is a rare, low-grade malignant vascular tumor [1–3]. It was first described as a distinct entity by Weiss and Enzinger in 1982 [4]. EHE occurs mostly in soft tissues of the extremities and various visceral organs (lung, bone, brain and intestine, etc.). Primary hepatic epithelioid hemangioendothelioma (HEHE) is a very rare type of malignant tumor first reported by Ishak et al in 1984 [5]. Clinical manifestations of hepatic epithelioid hemangioendothelioma (HEHE) are nonspecific, such as right upper quadrant pain, hepatomegaly and weight loss while many patients remain asymptomatic at diagnosis [2]. The duration of clinical symptoms ranges from 3 months to 2 years before the diagnosis of HEHE is made [3]. Laboratory examination shows that liver enzymes can be moderately elevated, but tumor marker levels (alpha-fetoprotein, carcinoembryonic antigen and cancer antigen 19-9) are usually normal. Therefore, clinical diagnosis of HEHE remains very difficult where imaging investigations, especially computed tomography (CT) or magnetic resonance imaging (MRI), play an important role in the diagnosis of HEHE.

To our best knowledge, current imaging studies on HEHE remained very limited, most of which, if any, were about sporadic cases and small case series. In this study, we retrospectively analyzed the CT and MRI features of histopathologically confirmed HEHE in 11 patients. We emphatically described imaging findings of HEHE, including the patterns of growth and patterns of contrast enhancement, so as to improve the understanding of the disease.

## Methods

### Patients

The study was conducted in accordance with ethical guidelines for human research and was compliant with the Health Insurance Portability and Accountability Act (HIPAA). As such, the study received IRB or ethical committee approval, and that written informed consent was obtained from all patients. The ethics approval was provided by The First Affiliated Hospital, Sun Yat-Sen University, China.

Eleven patients with HEHE, histopathologically confirmed by surgical excision or biopsy, were recruited at our centre from 2003 to 2013. Histopathologically, tumor cells of HEHE consist of epithelial and dendritic cells. Tumor cells grow along pre-existing sinusoids with intervening collagenous fibrosis. They typically exhibit intracytoplasmic lumina, containing erythrocytes. Immunohistochemistry stains are positive for Vimentin and CD34 [1].

Among these 11 cases, there were six males and five females, with ages ranging from 25 to 57 years old (mean, 37.8 years old).

### CT protocol

All patients underwent contrast-enhanced CT examinations (Aquilion 64, Toshiba). The contrast medium injected was iopromide (Ultravist, 300mgI/ml) with a dose of 1.5 ml/kg and a injection rate of 3.5–4.0 ml/s. Images were obtained separately at the arterial phase (34–37 s after injection), portal venous phase (60–70s after injection) and delayed phase (3 min–5 min after injection). The scanning parameters included: tube voltage, 120 kV; tube current, 250 mA; pitch, 0.9; matrix, 512 × 512; slice thickness, 0.5 mm.

### MR imaging

All MR examinations were performed on a 3.0 T scanner (Magnetom Trio, Siemens Healthcare Sector) with an eight-channel torso-array coil. All patients underwent breathing training to ensure imaging quality and were examined in supine position after a fasting period of 4 h. The scanning range covered from the dome of the diaphragm to the last plane of the liver. Unenhanced scanning included axial T1-weighted images (acquired matrix, 256 × 192; TR, 200 ms; TE, 2.2 ms) and axial T2-weighted images (acquired matrix, 256 × 192; TR, 6000 ms; TE, 80 ms). Dynamic contrast-enhanced scan was performed with a 3D-VIBE sequence (volume interpolated breath hold examination) with the following parameters: acquired matrix, 256 × 192; TR, 3.3 ms; TE, 1.1 ms; section thickness, 2 mm; intersection gap, 1 mm. The contrast medium was applied in terms of a bolus injection of Gd-DTPA with a dose of 2 mmol/ (kg body weight) and an injection rate of 2 ml/s. Dynamic images were obtained 15 s after injection. The other parameters of MRI scanning were: field of view, 380 × 380 mm; flip angle, 12°; time obtained, 16 s.

### Image analysis

All CT and MR images were reviewed independently by two experienced radiologists who were blinded to the identity of patients and their clinical outcome. They analyzed the images in terms of patterns of growth, characteristics of distribution, size, characteristics of density (sign) and patterns of contrast enhancement respectively. The recorded results were based on consensus. Comparison among the three groups of contrast enhancement were made using Kruskal-Wallis *H* test. The three pairwise comparisons were tested and Bonferroni’s adjustment was applied, the reported *P* values are two-sided and reflect Bonferroni’s adjustment. All statistical calculations were performed using SPSS software (version 17.0). Two-sided *P* values of less than 0.05 were considered to indicate statistical significance.

## Results

**Fig. 1:**
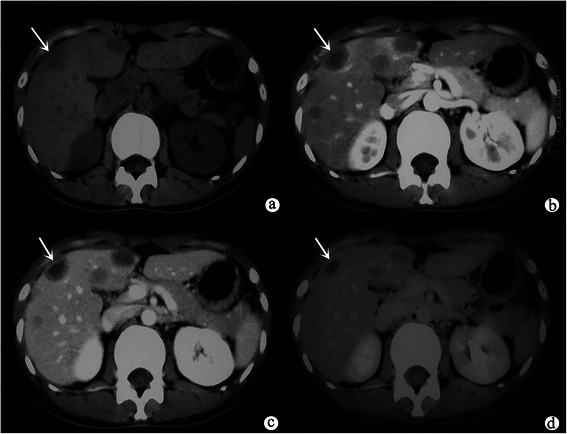
Axial unenhanced CT (**a**) showed that multiple nodular lesions (indicated by white and black arrow) were homogeneous and hypodense in the liver. Capsular retraction also could be seen (indicated by black arrow). Axial arterial phase (**b**) showed the lesion (indicated by the white arrows) was ring-like enhancement. Axial portal vein phase (**c**) showed the lesion (indicated by white arrow) was progressive enhancement. Axial delayed phase (**d**) the lesion (indicated by white arrow) was progressive enhancement

**Fig. 2:**
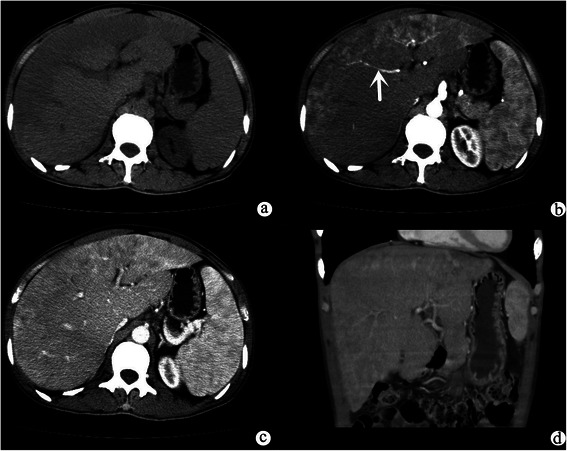
Axial unenhanced CT scan (**a**) showed lesions were homogeneous, lamellar, hypodense and subcapsular. Axial arterial phase (**b**) showed the lesions were heterogeneous enhancement, and a blood vessel tapered toward the lesions and was ended in the lesions (indicated by white arrow). Axial portal vein phase (**c**) showed the lesions were progressive enhancement. Coronal contrast-enhanced CT scan (**d**) showed the lesions were subcapsular

**Fig. 3:**
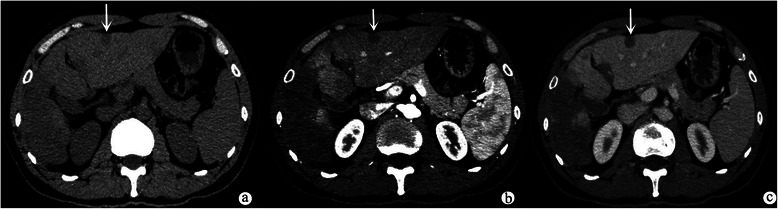
Axial unenhanced CT scan (**a**) showed lesion (indicated by the white arrow) was homogeneous, hypodense and subcapsular. Axial arterial phase (**b**) showed the lesion (indicated by the white arrow) was mild homogeneous enhancement. Axial portal vein phase (**c**) showed the lesion (indicated by the white arrow) was not progressive enhancement

Multiple lesions were found in the liver. According to the patterns of growth, these 11 cases can be divided into three types: nodular type, the lesions were circular or nodular (5/11, 45.5 %) (Fig. 1); coalescent type, the multiple lesions were peripheral subcapsular distribution with partial coalescence (1/11, 9.0 %) (Fig. 2); and mixed type, the lesions with both types mentioned above (5/11, 45.5 %) (Fig. 3). A total of 312 lesions were detected, and 214 (74.3 %) of them were subcapsular. Lesions were more frequently found in the right lobe (197/312, 63.1 %). The tumors ranged from 0.3 cm to 20 cm in size with 227/312 (72.8 %) lesions smaller than 2.0 cm, 53/312(17.0 %) between 2.0 cm and 3.0 cm, and 32/312(10.2 %) larger than 3.0 cm.

**Fig. 4:**
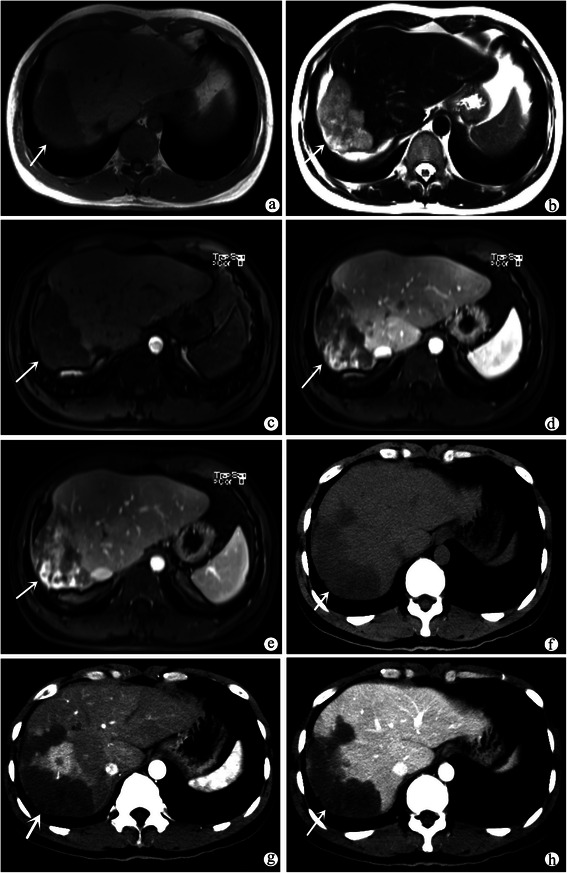
Axial unenhanced MRI scan showed lesions (indicated by the white arrow) were low signal intensity on T1WI (**a**) and high heterogeneous signal intensity on T2WI (**b**). The lesions (indicated by the white arrow) showed heterogeneous enhancement in the arterial phase (**c**), progressive enhancement in portal vein phase and in delayed phase (**d** and **e**). Axial CT images (indicated by the white arrow) were similar to MRI images (**f**, **g** and **h**)

All lesions were hypodense in appearance and round lower density were found within 10 lesions (<2 cm) on unenhanced CT scanning without calcifications. Five patients with MR examination had 61 lesions in MRI. All lesions demonstrated low signal intensity on T1 weighted images and high heterogeneous signal intensity on T2 weighted images compared to the normal liver parenchyma (Fig. 4).

In summary, the lesions showed three patterns of enhancement on both contrast-enhanced CT and MR images. The first pattern is mild homogeneous enhancement: mild homogeneous enhancements during the arterial phase, but no evidence of progressive enhancements during portal vein phase or delayed phase (223/312, 71.5 %) (Fig. 3). The second pattern is ring-like enhancement: peripheral enhancement in the arterial phase and progressive enhancement during portal vein phase and delayed phase (Fig. 1). The third pattern is heterogeneous delayed enhancement: heterogeneous enhancement during arterial phase and progressive enhancement during portal vein phase and delayed phase (Fig. 2). Lesions smaller than 2.0 cm mostly demonstrated mild homogeneous enhancement (214/227, 94.3 %); lesions measuring 2.0–3.0 cm showed ring-like enhancement (16/53, 30.2 %) and heterogeneous delayed enhancement (29/53, 54.7 %); and the lesions larger than 3 cm demonstrated heterogeneous delayed enhancement (26/32, 81.3 %). Retraction of the liver capsule overlying tumors was detected in six patients (6/11, 54.5 %) (Fig. 1). Six patients showed “lollipop sign” (6/11, 54.5 %), that is, the hepatic vein, portal vein and their branches are tapering toward the lesions and terminating at the edge of the tumor, which forms the appearance of a lollipop (Fig. 2). Capsular retraction and “lollipop sign” were found in lesions larger than 2.0 cm. No metastases or ascites were found in this cohort.

**Fig. 5:**
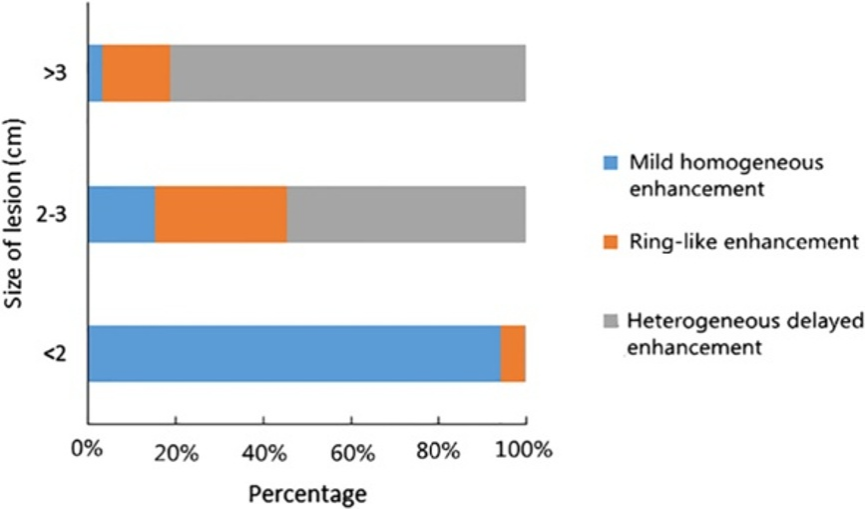
The relationship between the size of lesion and patterns of contrast enhancement

The imaging findings of all 11 cases are shown in Table 1. The relationship between the lesion size and the patterns of contrast enhancement is shown in Fig. 5. Comparison among the three patterns of contrast enhancement by using Kruskal-Wallis *H* test reported an *H* of 226 with *P* < 0.001 and two-sided *P* values were shown in Table 2.

**Table 1:** The imaging features of all cases with hepatic epithelioid hemangioendothelioma

Case No.	Growth pattern	Number of lesions	Capsular retraction	“Lollipop sign”	Patterns of contrast enhancement		
					Mild homogeneous enhancement	Ring-like enhancement	Heterogeneous delayed enhancement
1	nodular type	9	-	-	√	-	√
2	nodular type	35	√	-	√	√	√
3	mixed type	11	-	√	√	-	√
4	nodular type	16	√	-	√	√	-
5	mixed type	24	√	√	-	√	√
6	nodular type	105	-	-	√	-	√
7	mixed type	7	√	-	√	-	√
8	nodular type	19	-	√	√	-	√
9	coalescent type	1	√	√	-	-	√
10	mixed type	76	-	√	√	√	√
11	mixed type	9	√	√	√	-	√

**Table 2:** Two-sided *P* values

Groups	Mann-Whitney *U*	*Z*	*P*
Mild and Ring-like	1086.50	−13.141	<0.001
Mild and heterogeneous	168.00	−13.369	<0.001
Ring-like and heterogeneous	611.00	−2.546	0.022

### Pathology findings

**Fig. 6:**
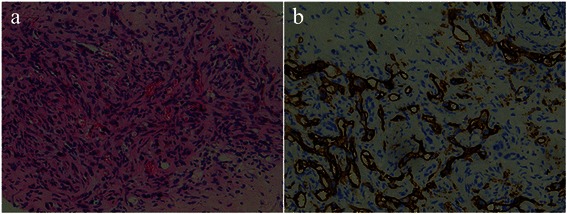
Micrograph (×200; hematoxylin-eosin stain) (**a**) showed some tumor cells presented intracellular vascular lumina with cytoplasmatic vacuoles containing erythrocytes. Immunohistochemistry (×200; CD 34 antibody stain) (**b**) showed positive results for CD34

Neoplastic cells in all 11 cases demonstrated presence of epithelial and dendritic cells with pleomorphic and polyhedral appearance; CD34 was positive in all patients (Fig. 6).

## Discussion

HEHE is a rare, low-grade malignant vascular tumor. The risk factors are currently unknown. It may be related to the use of oral contraceptive pills, chronic hepatitis B, excessive drinking and past history of chloroethylene exposure [1, 6]. The tumor is usually found in adults and shows a slight female predominance (male-to-female ratio, 2:3). The peak age of diagnosis is 30–40 years old [1, 3, 7]. HEHE can be divided into solitary lesion and multiple lesions in the liver. It has been reported that most of HEHE cases are characterized by multiple lesions, solitary lesion only accounts for 13 % – 18 % [1, 2]. All patients in the study were characterized by multiple lesions in the liver. Metastases have been reported in 27 %–37 % of patients, usually in the lung, and other common sites including regional lymph nodes, peritoneum, omentum, mesentery and bone. However, no metastases or ascites were found in the study.

Pathologically, HEHE tumor cells in our study demonstrated presence of intracytoplasmic lumina and erythrocytes with positive CD34 which were consistent with the findings obtained from previous reports,

Lesions of HEHE are more frequently subcapsular [8, 9], and 74.3 % of the lesions were subcapsular in our study. According to previous reports, there are two patterns of growth in the gross appearance of HEHE: the nodular type and the diffuse type [10]. However, three types were identified in our study, including nodular type, coalescent type and mixed type, in HEHE, in which the coalescent type has seldom been reported in previous reports. Nodular type (45.5 %) and mixed type (45.5 %) accounted for the vast majority of HEHE in the study. The simple coalescent type presented in only one case, however there were six cases (54.5 %) with coalescent growth. We consider that mild mass effect of coalescent type is a characteristic manifestation of HEHE. In other tumors the pattern of coalescent growth was rarely found, hence this pattern may be an important implication to the diagnosis of HEHE.

All lesions were hypodense on unenhanced CT. Calcification is considered as one of the common features seen in approximately 15 %–25 % of patients as suggested by previous reports, however, our findings showed inconsistent data. This may be due to the relatively small sample size (11) in our study. All lesions observed demonstrated low signal intensity on T1 weighted images and high heterogeneous signal intensity on T2 weighted images compared to the normal liver parenchyma.

Miller et al [11] reported capsular retraction as an important finding of HEHE. The pathological basis is hepatic fibrosis caused by the lesion and compensatory hypertrophy of unaffected hepatic segments [12–14]. In our study, six cases showed capsular retraction in lesions larger than 2.0 cm. This may be explained by that larger tumors are more likely to be located in the hepatic subcapsule or to cause local hepatic fibrosis. However, capsular retraction may also be one of the features seen in other benign or malignant liver lesions, such as cholangiocarcinoma and metastatic carcinoma. Therefore, capsular retraction remains an important finding but not a specific sign of HEHE.

Another important finding of HEHE is the “lollipop sign” as reported by Alomari et al in 2006 [15], who thought that “lollipop sign” was a characteristic finding of HEHE. Six cases showed “lollipop sign” in the study. “Lollipop sign” rarely occurs in most benign and malignant hepatic tumors, hence it can be considered as more characteristic finding of HEHE.

Dynamic contrast-enhanced scanning plays an important role in the diagnosis of HEHE. In general, as a vascular tumor, HEHE shows delayed enhancement in dynamic enhanced scanning. In this study, we found that the lesions showed three patterns of contrast enhancement, including mild homogeneous enhancement, ring-like enhancement and heterogeneous delayed enhancement. We also found that the patterns of contrast enhancement were closely related to the size of lesions. Smaller lesions (<2.0 cm) mostly showed mild homogeneous enhancement. With the enlargement of the lesions, HEHE could be characterized by multiple enhanced patterns. Lager lesions (>3.0 cm) mostly showed heterogeneous delayed enhancement. We consider that different patterns of contrast enhancement are related to pathological basis. Pathologically, the tumor tissues include epithelial and dendritic cells in variable proportions [1, 3, 16]. Larger tumor cells typically demonstrate presence of intracytoplasmic lumina containing erythrocytes, which resembles signet ring-like structures [17]. The peripheral tumor cells grow along preexisting sinusoids and terminal hepatic venules. Atrophic hepatocytes are obliterated. These may lead to heterogeneous enhancement of HEHE [1]. The presence of peripheral rich cellular zone and tissue edema may contribute to high density during enhanced scanning. The presence of abundant mucinous and stroma may contribute to the lack of central unenhanced areas [10, 18].

Based on our findings, HEHE could be discriminated from its differential diagnosis such as hepatic metastatic carcinoma, cholangiocarcinoma, and other liver vascular tumors like hepatic angiosarcoma or cavernous hemangioma.

The image features of hepatic metastatic carcinoma are more complicated. Similar to HEHE, this disease shows ring-like enhancement or nonspecific enhancement. However, the following tips could be applied in distinguishing hepatic metastatic carcinoma from HEHE: a known history of a primary malignancy; most commonly seen as peripheral enhancement lesions together with less common features such as delayed enhancement and invading blood vessels. Further more, HEHE lesions mostly demonstrate mild to moderate FDG uptake, while hepatic metastatic carcinoma commonly demonstrate intense FDG uptake. PET-CT could be used to detect the presence of metastatic tumor affecting distant organs to provide accurate staging.

For cholangiocarcinoma, it usually grows along the bile ducts. The adjacent bile ducts are nearly always dilated or are embedded by tumors. Invaded blood vessels are also shown. Cancer antigen 19-9 is usually elevated.

Other hepatic vascular tumors can be discriminated from HEHE, Hepatic cavernous hemangioma usually demonstrate more regular and obvious enhancement with similar appearances to arteries during arterial phase. On the other hand, hepatic angiosarcoma, a high-grade malignant vascular tumor, is often characterized by its irregular enhancement during arterial phase with enhanced nodular edge during portal vein phase or in delayed phase. Capsular retraction are usually not seen in hepatic vascular tumors.

## Conclusion

In conclusion, HEHE is rare, but it demonstrates several characteristics on imaging such as the presence of coalescent growth and “lollipop sign” to allow differentiation from other hepatic tumors. When interpreting these images, it should be kept in mind that the pattern of contrast enhancement are related to the size of lesions. Therefore, the pattern of enhancement can be variable on both MRI and CT, it can be used as a reliable indicator for the diagnosis of HEHE.

## Abbreviations

CT: Computed tomography
MRI: Magnetic resonance imaging
HEHE: Hepatic epithelioid hemangioendothelioma
EHE: Epithelioid hemangioendothelioma
HIPAA: Health Insurance Portability and Accountability Act
